# Genome-wide analysis reveals TET-and TDG-mediated 5-methylcytosine oxidation dynamics

**DOI:** 10.1186/1756-8935-6-S1-P75

**Published:** 2013-03-18

**Authors:** Li Shen, Hao Wu, Dinh Diep, Ana C D’Alessio, Alan Fung, Kun Zhang, Yi Zhang

**Affiliations:** 1 Harvard Medical School and Boston Childrens Hospital, Boston, USA

## 

Recent studies suggest that DNA demethylation can be achieved through ten-eleven translocation (Tet) family of DNA deoxygenates mediated oxidation followed by thymine DNA glycosylase (TDG) mediated excision and repair, but it is unclear to what extent such active demethylation processes take place. Here, we generated genome-wide distribution maps of 5-methylcytosine(5mC),5-hydroxymethylcytosine (5hmC), 5-formylcytosine (5fC) and 5- carboxylcytosine (5caC) in wild-type and Tdg-deficient mouse embryonic stem cells. We observe that the steady state 5fC and 5caC are preferentially detected at repetitive sequences in wild-type cells. Depletion of TDG causes marked accumulation of 5fC and 5caC at a large number of distal gene regulatory elements and transcriptionally repressed/poised gene promoters, suggesting that Tet/TDG- dependent dynamic cycling of 5mC oxidation states may be involved in regulating the function of these regions. Thus, comprehensive mapping of 5mC oxidation and BER pathway activity provides a promising approach for better understanding of DNA methylation and demethylation dynamics in development and diseases.

